# Structural Evolution Mechanism of Crystalline Polymers in the Isothermal Melt-Crystallization Process: A Proposition Based on Simultaneous WAXD/SAXS/FTIR Measurements

**DOI:** 10.3390/polym11081316

**Published:** 2019-08-06

**Authors:** Kohji Tashiro, Hiroko Yamamoto

**Affiliations:** Department of Future Industry-Oriented Basic Science and Materials, Toyota Technological Institute, Tempaku, Nagoya 468-8511, Japan

**Keywords:** isothermal crystallization, hierarchical structure, crystalline polymer, wide-angle X-ray scattering, small-angle X-ray scattering, infrared spectra, simultaneous measurement

## Abstract

Time-resolved simultaneous measurements of wide-angle X-ray diffraction (WAXD) and small-angle X-ray scattering (SAXS) (and FTIR spectra) were performed for various kinds of crystalline polymers in isothermal melt-crystallization processes, from which the common features of the structural evolution process as well as the different behaviors intrinsic to the individual polymer species were extracted. The polymers targeted here were polyethylene, isotactic polypropylene, polyoxymethylene, aliphatic nylon, vinylidene fluoride copolymer, trans-polyisoprene, and poly(alkylene terephthalate). A universal concept of the microscopically viewed structural evolution process in isothermal crystallization may be described as follows: (i) the small domains composed of locally regular but more or less disordered helical chain segments are created in the melt (this important information was obtained by the IR spectral data analysis); (ii) these domains grow larger as the length and number of more regular helical segments increase with time; (iii) the correlation among the domains becomes stronger and they approach each other; and (iv) they merge into the stacked lamellar structure consisting of the regularly arranged crystalline lattices. The inner structure of the domains is different depending on the polymer species, as known from the IR spectral data.

## 1. Introduction

The crystallization behavior of a crystalline polymer is indispensable information for the development of polymer materials with excellent physical properties [1,2,3,4,5,6,7,8]. The hierarchical structure of a crystalline polymer must be clarified from such various structural points of view, such as chain conformation, packing structure of chains in the crystal lattice, stacking structure of lamellae, and inner structure of a spherulite. However, studies to reveal the concrete hierarchical structural change in the isothermal melt-crystallization process have been limited in the literature. One of the most effective methods for revealing the hierarchical structure is the simultaneous and highly time-resolved measurements of the various types of experimental data. As reported in a series of papers [9,10,11,12,13,14], we developed the simultaneous measurement systems of two-dimensional wide-angle X-ray diffraction (WAXD), small-angle X-ray scattering (SAXS), and Raman/FTIR spectra. The WAXD data provide us with information on chain conformation and the packing mode of chains in the crystal lattice, as well as the amorphous state. The SAXS data reveal the aggregation structure of crystalline lamellae in the amorphous matrix. The vibrational spectral data tell us the local conformation, the interatomic interactions, and the thermal motions of polymer chains. In some cases, even the morphological information can be derived from the IR spectral data [15]. By using these simultaneous measurement systems, the structural evolution in the isothermal crystallization process from the melt was investigated for various kinds of crystalline polymers. The authors want to emphasize here that using a combination of the popular X-ray scattering method with the vibrational spectroscopic method, detailed and concrete structural images in the crystallization process can be provided, although this type of research has been quite limited so far. Let us examine one example to show the usefulness of such a combination of various kinds of data.

For example, the WAXD and SAXS data were combined with FTIR data, all of which were collected in the isothermal crystallization process of high-density polyethylene (HDPE) from the melt. These data allowed us to derive the concrete structure evolution in the crystallization process of this polymer [16,17,18,19]. Immediately after the temperature jump, the random coils in the molten state started to regularize locally into the short trans zigzag segments containing some gauche bonds, as known from the IR data analysis. These so-called disordered-trans segments gathered together and finally transformed into the regular orthorhombic crystal phase, as known from the WAXD data. The SAXS data revealed the formation of lamellae. Regarding the growth of these lamellae, the new lamellae, or the daughter lamellae, started to appear among the original mother lamellae. The thus experimentally derived structural image of the intermediate phase of HDPE is important in regards to two points: (i) the microscopically viewed structural details in the early stage of the isothermal crystallization are suggested mainly by the IR spectral data; and (ii) the existence of the conformationally disordered intermediate phase is pointed out concretely, which is an important hint for the discussion of the lamellar growing process as traced by the WAXD/SAXS measurements. Of course, this type of image can easily be speculated without any experimental data, but it must be emphasized that such an ambiguous speculation has been confirmed for the first time by the experimental data. The abovementioned point (ii) is consistent with the lamellar growth model proposed by Strobl et al. [20,21], that the random coils in the melt are adsorbed on the front surface of the growing lamella and they are regularized to the intermediate region and that once the size of the intermediate region is beyond a critical point, the intermediate region transforms into the thermodynamically stable orthorhombic crystal lattice consisting of the long trans-zigzag segments. Many papers adopted their model for a qualitative interpretation of the structural change in the crystallization process. In such a point, their model looks to be generalized as a universal model for the description of the lamellar growing process in the crystallization of a polymer. Is it really correct to always apply this model to any kind of crystalline polymer? After thoroughly searching the literature, the explicit experimental evidence showing the existence of such a structurally disordered intermediate phase in the isothermal crystallization process from the melt was reported only for the abovementioned HDPE case, though many speculations were presented in the literature. It seems quite dangerous to apply this model so easily to any kind of polymer as it is. We have to quote the words of Dr. Keller [22], who warned of the easy generalization of chain folding mechanisms in the controversial discussions of the 1970s: “We have to notice that the discussion has been made only for polyethylene”. Besides, most of the papers only analyzed the WAXD and/or SAXS data to reveal the formation of the crystalline lattice, the stacked lamellar structure, etc., but the molecular chain state in the much earlier stages of crystallization has not yet been revealed on the experimental side [1,2,3,4,5,6,7,8]. For this purpose, the vibrational spectroscopy plays an important and indispensable role.

Over the past decades, the present authors have collected WAXD, SAXS, and vibrational spectroscopic data on the crystallization processes of various kinds of polymers by developing the simultaneous measurement systems, from which the characteristic structural regularization features were extracted concretely. The present review summarizes these experimental data and tries to extract the similarities and differences in the structural evolution processes among these various polymers. Then, we propose a structural evolution model to describe the crystallization process viewed from the various hierarchical structural points. This paper may be assumed as a warning against the easy universalization of one limited model.

## 2. Experimental Section

### 2.1. Polymers

The crystalline polymers used in the isothermal crystallization experiments were isotactic polypropylene (*it*-PP), Nylon 1010, trans-1,4-polyisoprene (PI), high-density polyethylene (HDPE), polyoxymethylene (POM), vinylidene fluoride-trifluoroethylene copolymers (VDF/TrFe = 73/27 molar ratio), and poly(alkylene terephthalate). The detailed characterizations of these samples are referred to in the individual papers.

### 2.2. Measurement Systems

The isothermal crystallization experiments were performed at several synchrotron beam lines as well as in the laboratory. The synchrotron facilities used were the Photon Factory (KEK, Tsukuba, Japan) beamline (BL) 10C, the SPring-8 (Harima, Japan) BL40B2, BL40XU, and BL03XU, the PAL (Pohang accelerator Laboratory, Pohang, Korea) BL-SAXS-4C, and the Aichi Synchrotron Radiation Center BL8S3. The wavelength of the incident X-ray beam was 1.5, 1.0 or 0.675 Å.

**Figure 1:**
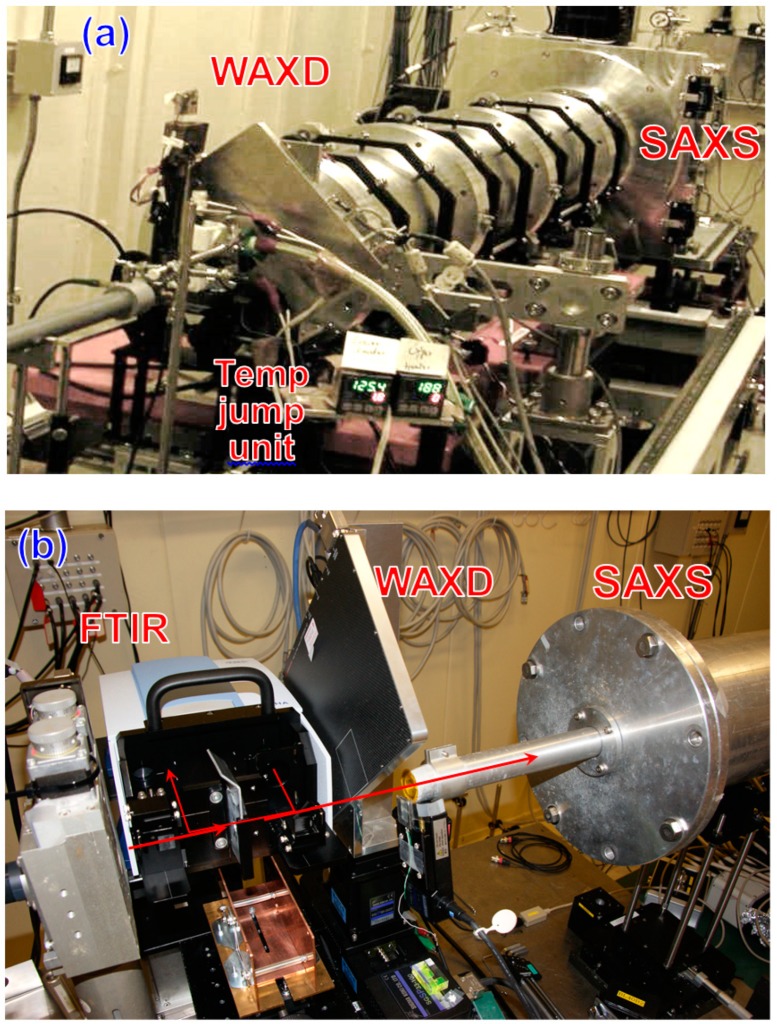
Simultaneous measurement systems in the synchrotron facilities. (**a**) WAXD and SAXS; (**b**) WAXD, SAXS, and FTIR spectra. In these two cases, the detectors were a flat panel (WAXD) and an image-intensifier-CCD (SAXS).

**Figure 2:**
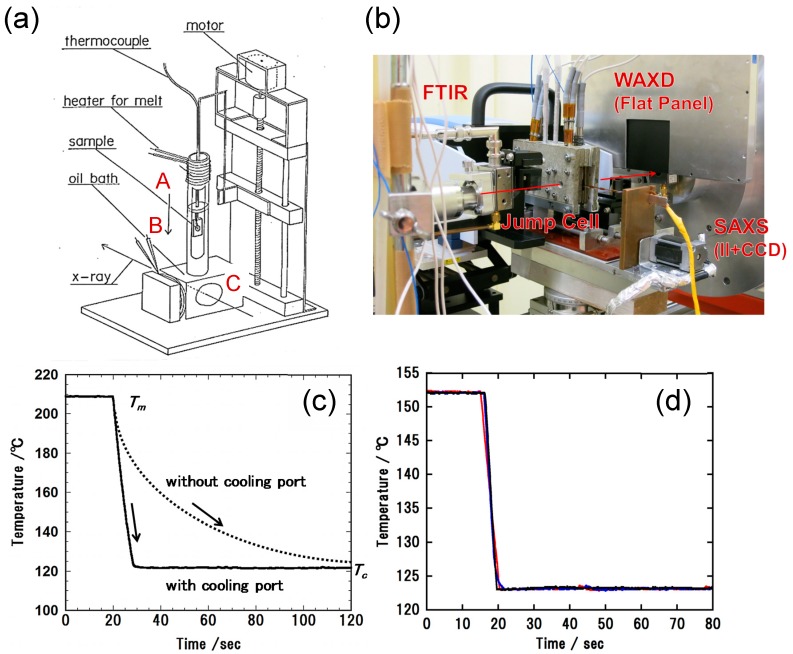
Temperature jump cells. (**a**) Manually operated system (the sample holder is moved vertically) and (**b**) computer-controlled system (the sample holder is moved horizontally from position (A) to (B) to (C)). (**c**) Temperature change in the sample in the temperature jump process with and without the cooling port at position (B) and (**d**) the reproducibility of the temperature change observed for the system (**b**).

(1) Simultaneous Measurement of WAXD and SAXS: The WAXD and SAXS data were collected simultaneously by setting the two detectors at different distances from the sample cell, as illustrated in Figure 1a [14]. The detectors used were a flat panel detector (C9728-DK, Hamamatsu Photonics K.K., Shizuoka, Japan) for the WAXD data and an image-intensified CCD detector (C4742-98, Hamamatsu Photonics K.K.) for the SAXS data. The exposure time was 1–10 s per shot.
(2) Simultaneous Measurement of WAXD/SAXS/FTIR: The WAXD/SAXS data were collected using a similar system as illustrated in Figure 1b. The detectors used were a flat panel for WAXD data and an image-intensifier CCD camera or Pilatus 100k (Dectris, Baden-Daettwil, Switzerland) for SAXS data. A miniature FTIR spectrometer (Bruker α) was set around the sample cell to measure the transmission-type IR spectra [12]. The WAXD and SAXS data were collected at every 1–10 s. The FTIR spectra were collected at the resolution power of 2 cm^−1^ at every 7 s.
(3) Temperature Jump Cell: In the isothermal crystallization experiment, the temperature of a sample must be changed from the melting temperature (*T*m) to the crystallization temperature (*T*c) as quickly as possible and kept at *T*c as stably as possible. For this purpose, the temperature jump cells were developed. The jump cell of the first generation was the manually operated system illustrated in Figure 2a [14]. A sample was put into a small metal holder. The holder was first set at position (A) and was heated above *T*m for a fixed time to melt the sample. Then, the holder was immediately moved to position (B), where the sample holder was stopped for a while, during which the air was blown to the sample in order to erase the heat stored in the sample. As a result, the sample temperature decreased steeply to a value between *T*m and *T*c, as detected by a thermocouple embedded inside the sample. Then, the sample holder was moved quickly to position (C) at the temperature *T*c. The temporary stop at position (B) is key to producing a sharp temperature jump.

The temperature jump cell of the second generation is of the computer-controlled type, which was produced by modifying the original first-generation system. The digital data of the sample temperature were recorded by a computer, which controls the timing to move the sample holder from position (A) to (B) and (C) so that the most ideal change in temperature can be attained. Figure 2b shows a snapshot of the new temperature jump cell. The actually obtained trace of temperature is shown in Figure 2c. As shown in Figure 2d, the quick and stable temperature change was successfully attained with a good reproducibility. The temperature jump rate was about 600–2000 °C/min (dependent on the sample thickness) and the stability of temperature at *T*c was ±0.05~0.1 °C.

### 2.3. X-ray Scattering Data Analyses

The 2D WAXD ring patterns were converted to the 1D diffraction profiles by the total integration at a constant 2θ internal. The integrated intensities of the individual diffraction peaks were evaluated after the curve separation into the components. The 2D SAXS patterns were converted to the 1D patterns in a similar way.

The thus obtained 1D SAXS pattern profiles were found to be analyzable quantitatively on the basis of the three different theoretical treatments: the scattering by an isolated domain; the scattering from the correlated domains; and the scattering by the stacked lamellar structure. Of course, these concepts were abandoned whenever the data treatments could not be succeeded reasonably.

#### 2.3.1. Domains

As revealed by the IR spectral data, immediately after the temperature jump, the random coils in the melt changed their local conformation to the partially regular form. For example, *it*-PP forms the short helical segments in the melt after the temperature jump [23,24]. Other polymers examined here were found also to show the similar regularization of the chain conformation, as revealed by the IR data analysis. Such a local structural regularization of random coils may reflect on the SAXS profile. By taking into consideration these IR spectroscopic data as the quite important basic supports, the SAXS data collected immediately after the temperature jump were tried using a model of an isolated spherical domain with a radius of gyration *R_g_*. Usually, the quantitative estimation of *R*_g_ is carried out conveniently using a so-called Guinier plot: *I*(*q*) = *I*(0)exp(−*R_g_*^2^*q*^2^/3) where *I*(*q*) is the SAXS scattering intensity at the scattering angle 2θ or the scattering vector *q* = (4π/λ)sin(θ) [25,26,27]. This approximated equation can be used only for the SAXS data satisfying the condition *q* << 1/*R_g_*. However, in the actual SAXS experiment, the satisfaction of this condition is quite hard, since a relatively large beam stopper is set to avoid the damage of the detector by an incident direct X-ray beam and also to reduce the strong parasitic scattering originated from the slit system. Starting from the SAXS intensity equation of an isolated particle, the following approximated equation can be derived [25].

(1) $$I(q)\;=\;4\pi \int_{0}^{\infty }\gamma (r)[\sin(qr)/(qr)]r^{2}dr\;\approx \;I(0)[\;1\;–\;(M_{4}/6M_{2})D^{2}q^{2}\;+\;(M_{6}/120M_{2})D^{4}q^{4}]$$

where *γ*(*r*) is the correlation function and *D* is the longest length of the particle and is equal to the radius of a spherical model. *M*_k_ is the *k*th moment of the correlation function *γ*(*r*).

(2) $$M_{k}\;=\;D^{-(k+1)}\int_{0}^{D}r^{k}\gamma (r)dr$$

The approximated Equation (1) is said to be applicable in a wide *q* range of *q* ≤ 2*R*_g_^−1^. The observed intensity *I*(*q*) is fitted using Equation (1), from which the three coefficients are estimated. The *R_g_* is calculated using the first and second coefficients in Equation (1) as below.

(3) *R*_g_ = (*M*_4_/2*M*_2_)^1/2^*D*

#### 2.3.2. Correlated Domains 

The domains may gather together to form an aggregation of a high-density *ρ*. As a result, these aggregations are dispersed in a system with a correlation. The corresponding SAXS signal may be expressed using the correlation function with a form exp(−*r*/*ξ*). The result is given as follows [28].

*I*(*q*) = *A*/(1 + *ξ*^2^*q*^2^)^2^ (4)

where *A* is a constant and *ξ* is a correlation distance among the higher-density regions. In the actual data fitting, the data in the *q* range of 10^−3^~10^−2^ Å^−1^ was used to avoid the contribution of the SAXS components from the newly generated domains and the WAXD components due to the inner structure of the regions.

#### 2.3.3. Stacked Lamellae 

**Figure 3:**
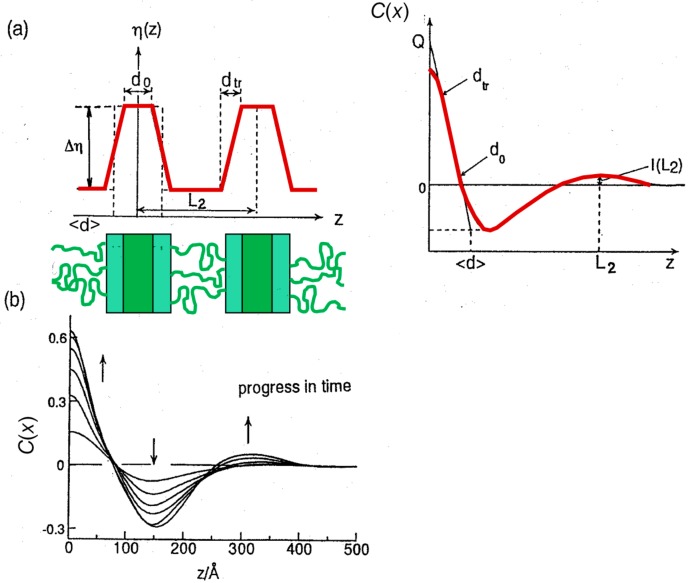
(**a**) Correlation function of the 1D stacked lamella structure and the relationship with the actual structural parameters and (**b**) the time dependence of the correlation function in the isothermal crystallization process of *it*-PP.

For crystallization, the stacked lamellar structure may be formed, which has a thickness *d* and a long period *LP* [25,26,27,28,29]. The SAXS signal originating from such a stacked lamellar structure was analyzed using the 1D lamellar correlation function *C*(*x*):

*C*(*x*) = <[*η*(*x* + *x*’) − <*η*>)]*[*η*(*x*’) − <*η*>]> = (2/π)∫*q*^2^*I*(*q*)cos(*qx*)d*q* (5)

where *η*(*x*) is the electron density at position *x* along the normal direction to the stacked lamellar plates, and <*η*> is the ensemble average. The *LP* and averaged lamellar thickness <*d*> are obtained from the points of *C*(*x*), as illustrated in Figure 3a. One example of the actually obtained *C*(*x*) is shown in Figure 3b.

## 3. Results and Discussion

### 3.1. Isotactic Polypropylene

**Figure 4:**
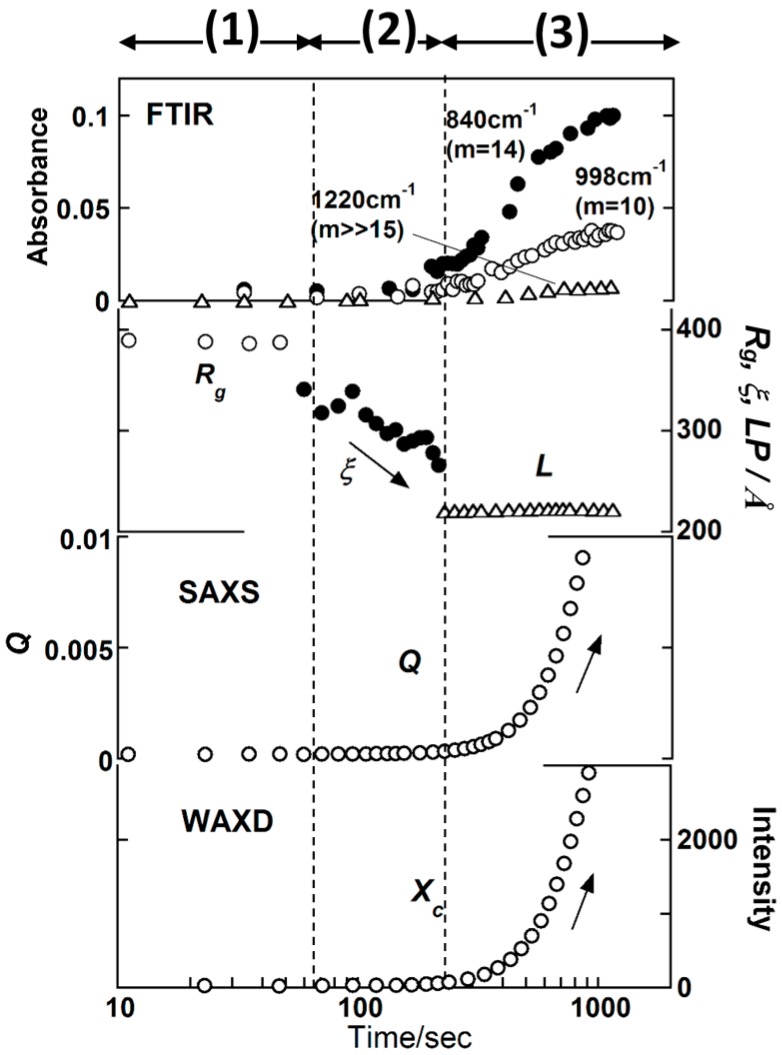
Time dependence of the IR band intensity, the SAXS structural parameters, and the WAXD crystallinity *X*c estimated in the isothermal crystallization process of *it*-PP, where the degree of super cooling ∆*T* = *T*_∞_ − *T*_c_ = 61 °C for the equilibrium melting temperature *T*_∞_.

In the previous papers [23,24], the WAXD and SAXS data of *it*-PP were measured simultaneously in the isothermal crystallization process from the melt. These data were combined with the separately measured FTIR data to discuss the structural evolution in the crystallization process. However, in order to adjust the crystallization conditions perfectly, the simultaneous measurement of these three data is needed. The newly obtained data are shown in Figure 4. These data were quantitatively analyzed by the methods mentioned in the preceding section. The result is essentially the same as that reported in previous papers. As already known [30], the IR band can be detected for the first time when the helical length is beyond a critical value, which is expressed as *m* or the number of monomeric units forming a critical helical segment. As seen in Figure 4, the 998 and 840 cm^−1^ bands with the short critical sequence length (*m* = 10 and 14, respectively) appeared at first [23,24]. After some delay, the 1220 cm^−1^ band with the longer critical sequence length (*m* ≫ 15) started to appear. These observations clearly indicate the growth of regular helical segments in the melt. In the time region (1) of the appearance of short helical segments, the SAXS data could be analyzed well by the domain model with *R*_g_ of about 400 Å. This correspondence between the IR and SAXS observation indicates that the domain consisted of regular but short helical segments. In the time region (2), the helical segments grew further, as seen from the increase in the IR band intensity. In parallel, the correlation length *ξ* among the neighboring domains, estimated using Equation (4), became shorter with the passage of time. The *ξ* value was found to approach the *LP* value estimated from the SAXS correlation function. In this time region, the crystalline peaks started to appear in the WAXD pattern. The invariant *Q*, which was obtained from the SAXS data analysis as shown in Figure 3, and the degree of crystallinity *X*_c_ estimated from the WAXD data, started to increase at the same time.

**Figure 5:**
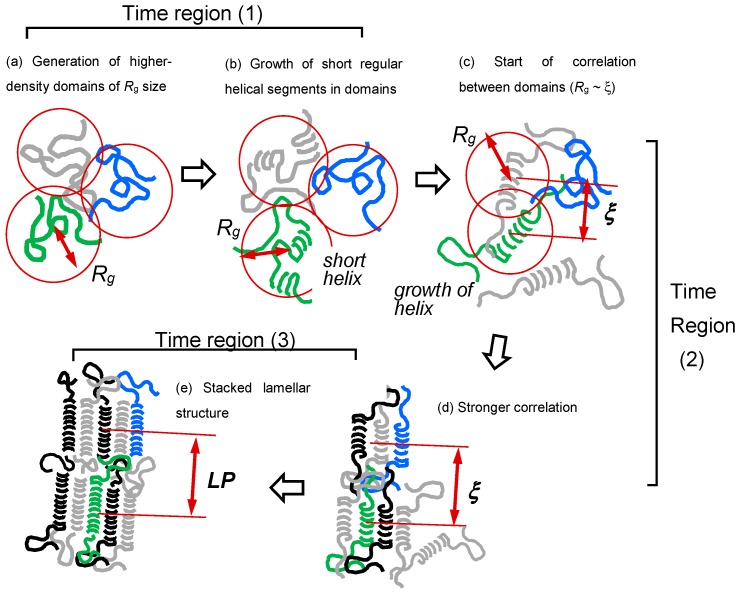
Illustration of a structural evolution process in the isothermal melt-crystallization of *it*-PP. (**a**) Higher-density domains of *R*_g_ size are generated in the melt, (**b**) the short regular helices are generated in the domains, (**c**) and these domains start to correlate with each other. The correlation length may be defined in the various directions, among which the shortest correlation length (*ξ*_min_) can be detected in the normal SAXS measurement. The SAXS components corresponding to the longer correlation are hidden by the beam stopper. (**c**,**d**) The *ξ*_min_ is converged finally with the averaged *LP* among the neighboring lamellae [36].

The structural evolution derived from these data analyses is illustrated in Figure 5. The relatively regular helical segments were generated in the melt and formed the domain of about 400 Å. These domains approached each other and gathered together to create the stacked lamellae of about a 220 Å long period.

### 3.2. Polyoxymethylene

**Figure 6:**
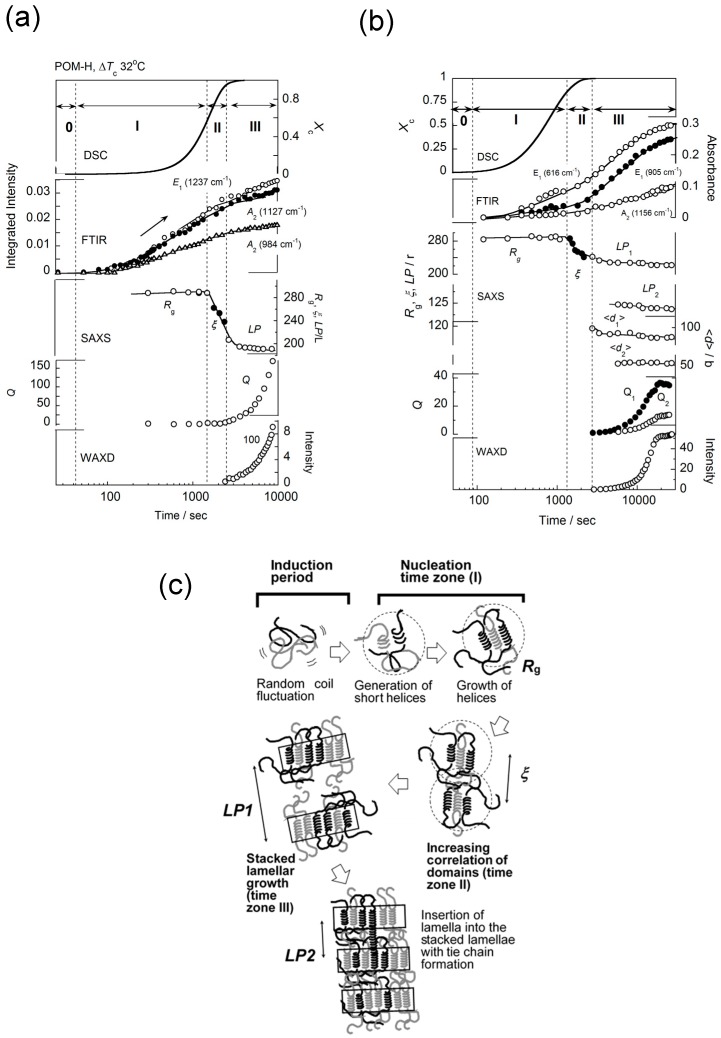
(**a**,**b**) The time dependence of the structural data collected in the isothermal crystallization process of the POM-H and POM-D samples, respectively. (**c**) The illustration of the structural evolution, where the insertion of daughter lamellae is also included [32].

POM crystallizes relatively fast. As shown in Figure 6a, the IR bands at 1237, 1127, and 984 cm^−1^, which have short critical sequence lengths of *m* = 1~5, were found to appear quite rapidly, indicating the growth of regular helical segments in the very early stage of the domain formation stage [31,32,33]. The domains consisting of these short helices continued to grow for a comparatively long time. These domains approached each other and grew finally to the stacked lamellar structure. Similar behavior was also observed for the deuterated POM (Figure 6b). In this case, in addition to the common phenomenon of the domain growth into the lamella, the lamellar insertion phenomenon was observed. That is, the mother lamellae were generated at first in the early stage of time region (III). After that, the daughter lamellae, with a half-long period as that of the mother lamellae, started to appear in the amorphous region among the original mother lamellae (see Figure 6c) [34,35].

### 3.3. trans-1,4-Polyisoprene

**Figure 7:**
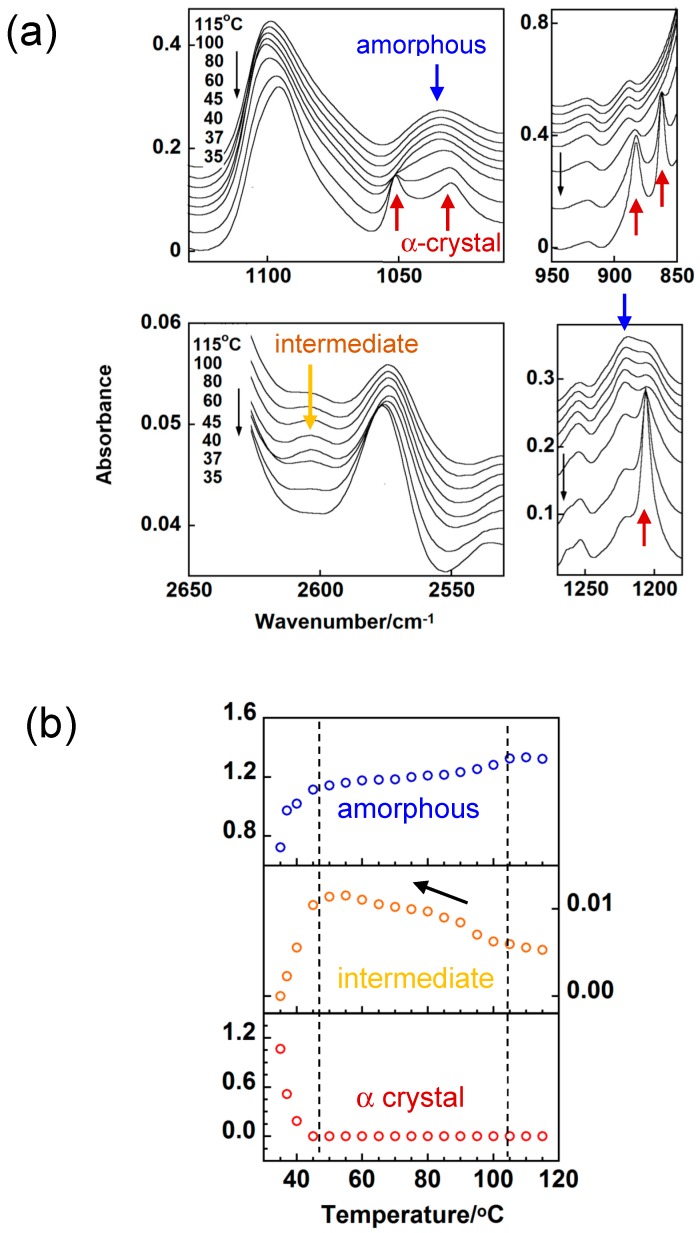
Temperature dependence of (**a**) IR spectra and (**b**) integrated intensity of the amorphous, intermediate and crystalline (α) bands of trans-1,4-polyisoprene measured in the cooling process from the melt [36].

The appearance of an intermediate phase can be detected clearly for this polymer [36,37]. As shown in Figure 7, where the temperature-dependent intensity change was plotted for the various IR bands in the continuous cooling process, the band at 1205 cm^−1^ appeared when the molten sample was cooled to about 45 °C. This band comes from the crystalline α phase. Many other α bands were also observed at 45 °C. The band at 2603 cm^−1^ showed different behavior; this band was observed in the molten state, but the intensity increased continuously before the appearance of the crystalline α bands, suggesting the existence of an intermediate phase between the melt and the α phase.

**Figure 8:**
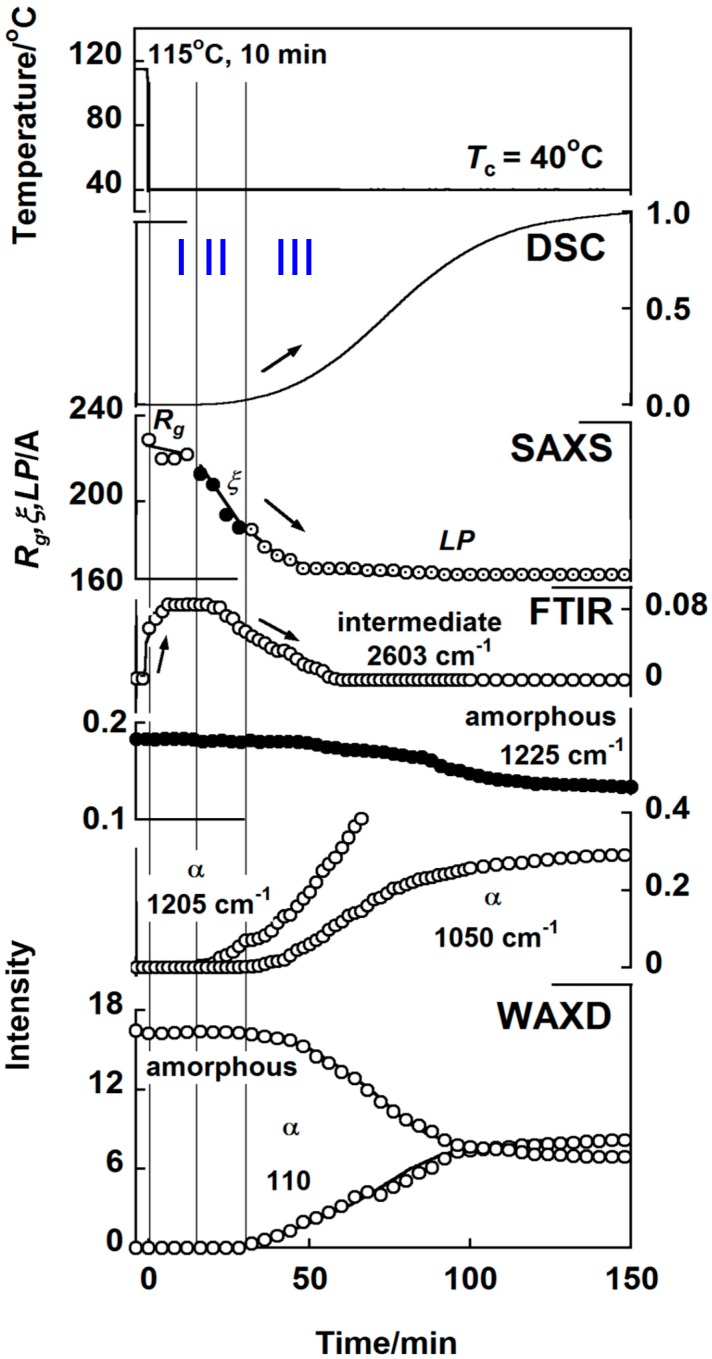
Time dependence of the various structural parameters collected for trans-1,4-polyisoprene in the isothermal crystallization from the melt. The intermediate phase started to appear immediately after the temperature jump and existed for a while until the crystalline α phase was created and increased its amount [36].

The isothermal crystallization process was traced in the temperature jump experiment at 40 °C, as shown in Figure 8. Immediately after the temperature reached the crystallization temperature, the 2603 cm^−1^ band started to appear. In the SAXS data, the domains with *R*_g_ of approximately 240 Å were detected in the early stage. After the correlation among the domains increased in the time region (II) and approached the crystalline state in the time region (III), the 2603 cm^−1^ band intensity decreased and the α phase bands started to appear. In parallel, the WAXD and SAXS peaks of the α form appeared. The two α IR bands at 1205 and 1050 cm^−1^ appeared at different timings, being originated from the difference in the critical sequence lengths. In this way, trans-1,4-polyisoprene crystallizes first in the intermediate phase, which disappears totally when the α crystalline phase appears as the regular phase.

### 3.4. High-Density Polyethylene

**Figure 9:**
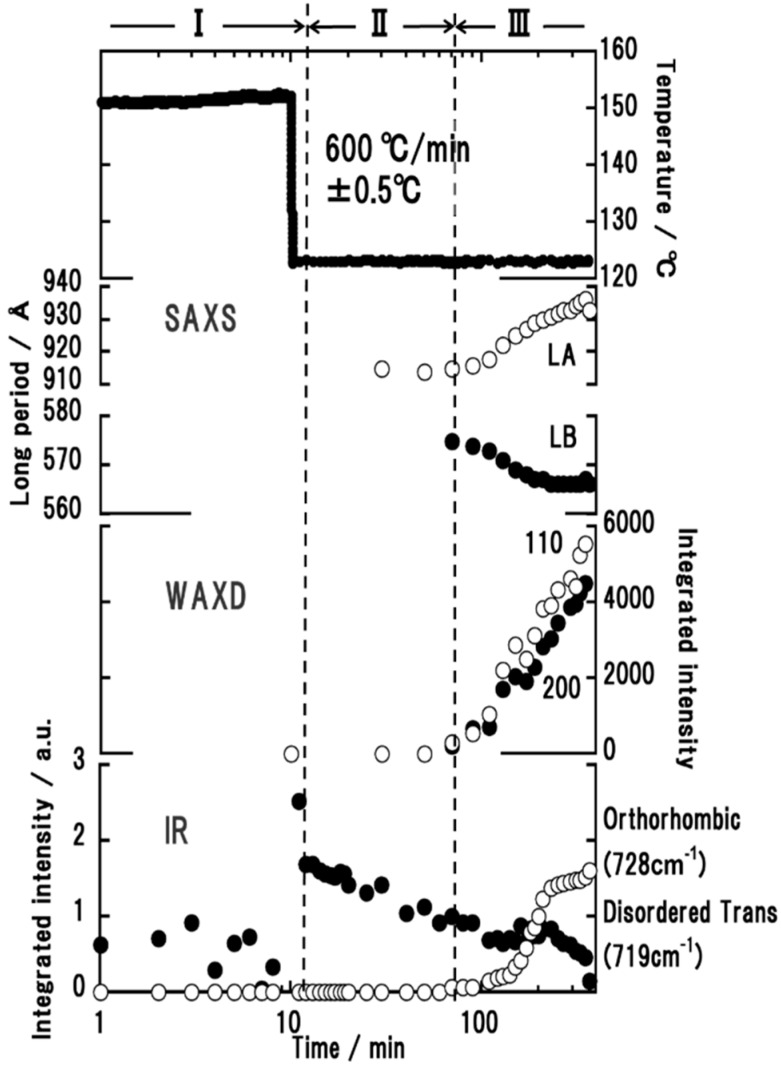
Time dependence of SAXS, WAXD, and IR data collected in the isothermal crystallization of high-density polyethylene.

**Figure 10:**
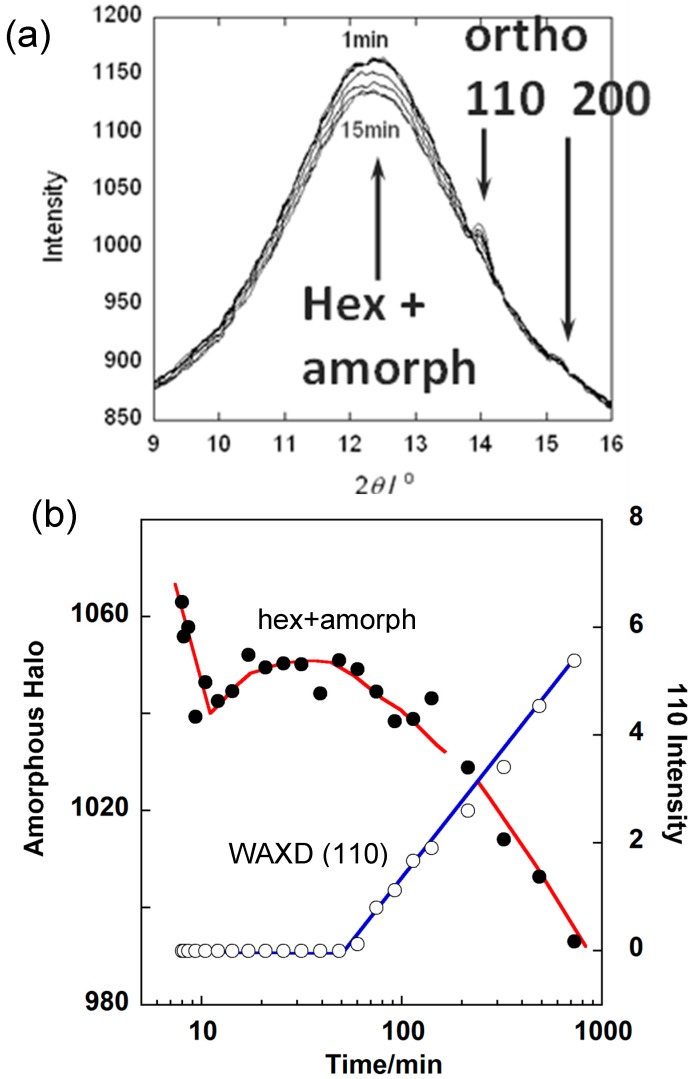
Time dependence of (**a**) WAXD profile and (**b**) the intensity of the amorphous halo and (110) reflection obtained in the isothermal crystallization of high-density polyethylene.

The crystallization rate of HDPE was quite high and the detection of the domains in the earlier stage was difficult in the SAXS measurement. As shown in Figure 9, the appearance of the stacked lamellae (LA) was detected in a later stage of the time region (II). The long period was approximately 900 Å. In the time region (III), the daughter lamellae (LB) started to appear with almost half as long a period as the mother lamellae. In this time region, the orthorhombic WAXD peaks (110 and 200) started to appear. As for the IR spectral data, in the time region (II), the so-called disordered trans band at 719 cm^−1^ increased in intensity, which should correspond to the conformationally disordered hexagonal phase [16,17,18,19]. As pointed out above, the mother lamellae were detected in parallel in the SAXS measurement. All of these data suggest that, in the time region (II), the mother lamellae consisted mainly of the hexagonal phase with the conformationally disordered chain segments. The WAXD data should show the peak of the hexagonal phase clearly in order to confirm this conclusion [18]. However, as shown in Figure 10a, the diffraction angle region predicted for the 100 hexagonal peak was covered with a strong amorphous halo, making it difficult to judge the existence of the hexagonal phase peak. Then, the integrated intensity of the whole broad peak was evaluated, the intensity of which was found to change at the two stages. The broad peak intensity started to decrease in the time region (II) and plateaued for a while. Then it decreased steeply (Figure 10b); at this moment the orthorhombic peaks started to appear. This behavior strongly suggests the presence of the intermediate hexagonal phase before the appearance of the orthorhombic phase. This X-ray observation was consistent with the FTIR data. With the passage of time, the intensity of the disordered trans IR band decreased gradually, but it was detected even in the time region (III). The 728 cm^−1^ band, intrinsic to the orthorhombic phase, increased the intensity and coexisted with the disordered trans band.

**Figure 11:**
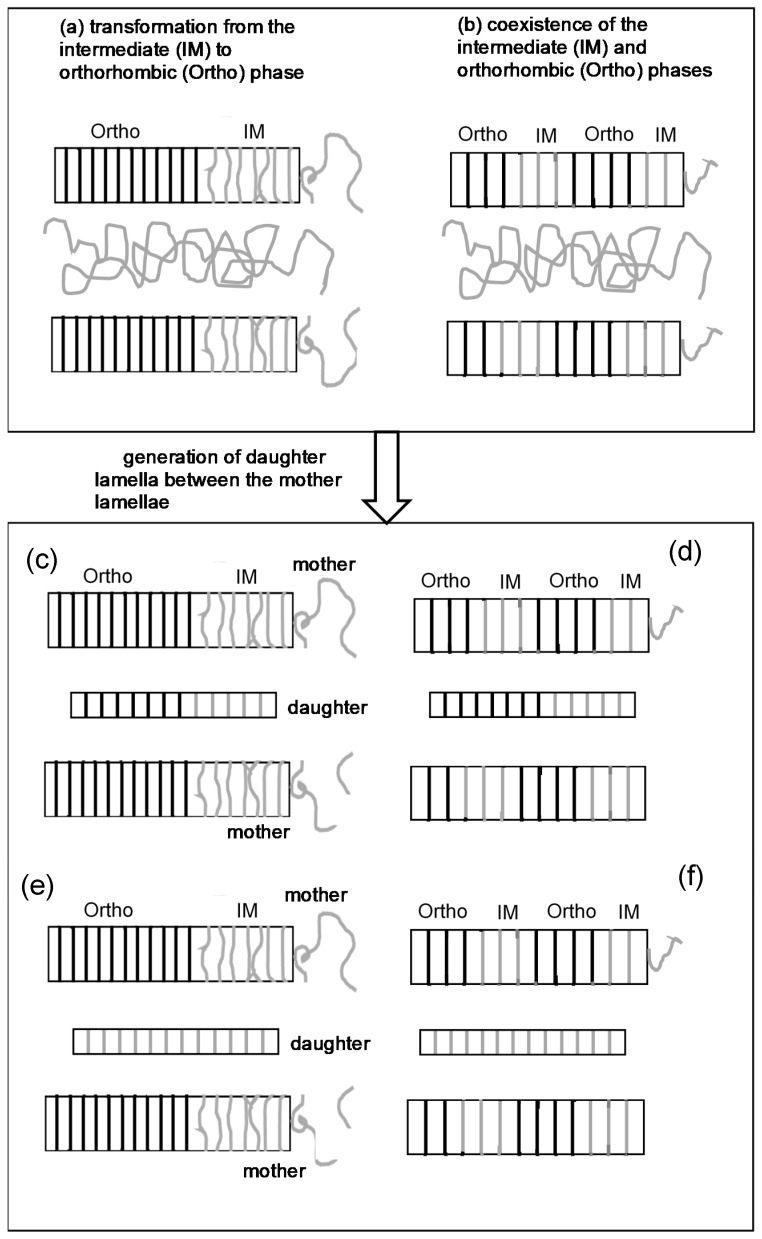
(**a**,**b**) The distribution of the intermediate and regular crystalline phases in the growing lamella. (**c-f**) The generation of the daughter lamella between the original mother lamellae.

In this way, the hexagonal phase transforms to the structurally regular orthorhombic phase. The daughter lamellae started to appear in the amorphous region and coexisted with the mother lamellae. Even after the several tens of minutes, where the orthorhombic phase increased appreciably, the signal of the intermediate state was detected, as known from the observation of the disordered-trans IR band. Besides, the WAXD 110 and 200 peaks increased the intensity gradually, not sharply. As shown in Figure 11, one model satisfying these observations may be illustrated in such a way that the disordered lamellae grew still in the time region (III), and they were regularized to the orthorhombic phase. Strobl et al. [20,21] imagined that the intermediate state might exist in the front face of the growing lamella, not the direct regularization of the amorphous random coils to the orthorhombic phase immediately after the adsorption of chains on the lamellar surface [20,21]. However, it is still ambiguous whether the intermediate phase generates only at the front face or it remains inside the regular lamellar plate and coexists with the orthorhombic phase (Figure 11b). The same question might be invoked also for the daughter lamellae. There are four possibilities regarding the daughter lamellae: they are generated in the amorphous region among the mother lamellae as the intermediate phase and transforms to the orthorhombic phase in a later stage (Figure 11c,d), or they exist totally as the intermediate phase (Figure 11e,f).

### 3.5. Nylon 1010

**Figure 12:**
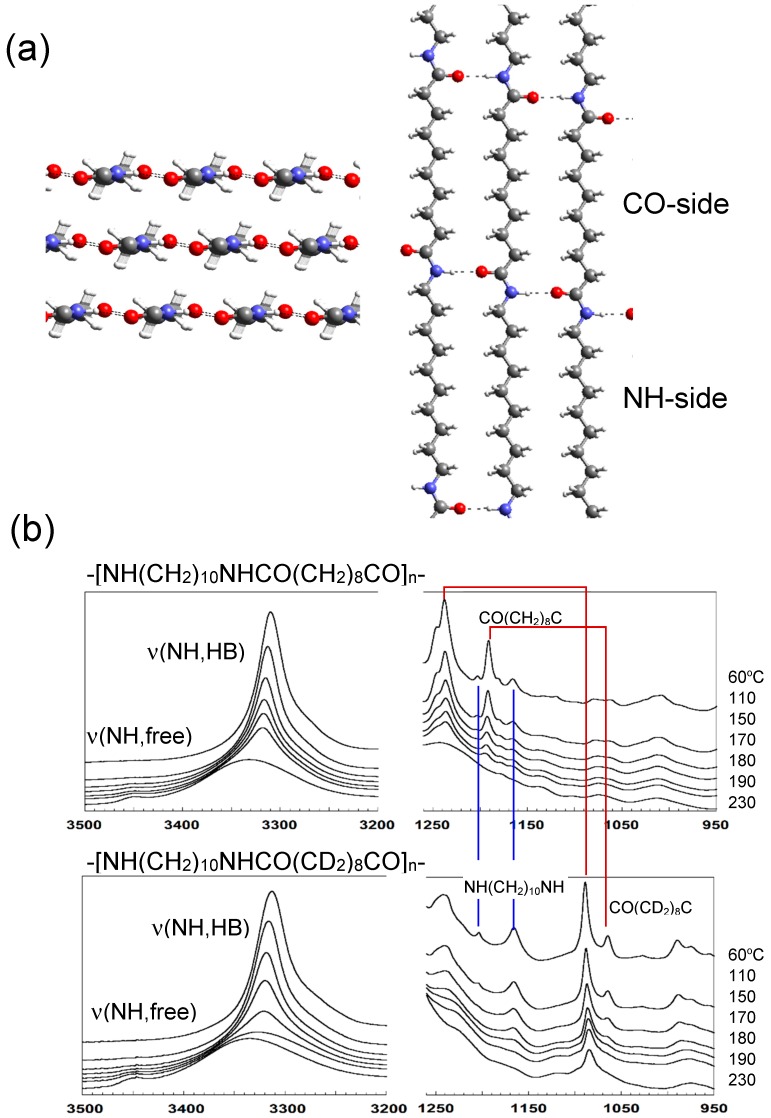
(**a**) Crystal structure of Nylon 1010. (**b**) Temperature dependence of IR spectra of nylon 1010 with normal methylene sequences and with the deuterated methylene sequences in the CO side. The band position was seen to shift toward the lower frequency side by the deuteration.

Aliphatic nylon consists of the alternate arrangement of methylene segments and amide groups along the chain axis. In the crystal lattice, the sheets, which are constructed by the parallel packing of zigzag chains linked by the intermolecular hydrogen bonds, are stacked together by the weaker van der Waals interactions, as illustrated in Figure 12 [38]. For the study of the structural evolution process from the melt, we need to know the conformational regularization of the chains, the arrangement of the methylene segmental parts, and the formation of hydrogen bonds among the amide groups in addition to the generation of the lamellar sacking structure. In order to trace the conformational regularization, the nylon sample with relatively long methylene segments is better because of the easier detection of a series of the so-called progression bands [39,40,41]. At the same time, the melting point is lower for such an aliphatic nylon with longer methylene segments and the thermal degradation is depressed more effectively. We chose nylon 1010, –(–NH(CH_2_)_10_NHCO(CH_2_)_8_CO–)*_n_*– for the crystallization study. Besides, it is better to distinguish the behavior of the two methylene segments of the monomeric unit. Then, the deuterated methylene segment was introduced as the CO-side methylene units: –(NH(CH_2_)_10_NHCO(CD_2_)_8_CO)*_n_*– [13,42]. As shown in Figure 12b, the IR CH_2_ bands shifted before and after the partial deuteration, making it easier to trace the behaviors of these two different types of the methylene segments. For example, this figure shows the temperature dependence of the IR spectra in the heating process, where the methylene bands of the NH side disappeared at a lower temperature than those of the CO side. The thermal mobility of the NH-side methylene segments seems more active than that of the CO-side methylene part [39,40,41].

**Figure 13:**
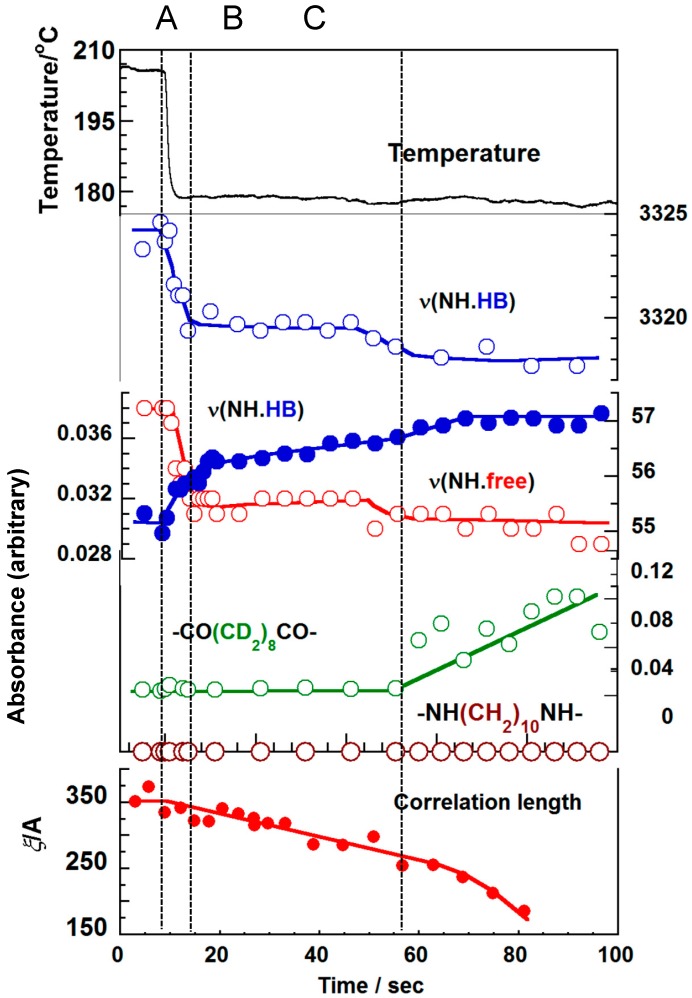
Time dependence of the various data measured for the partially-deuterated nylon 1010 sample in the isothermal crystallization from the melt [13].

Figure 13 shows the various experimental data collected for this partially deuterated nylon 1010 sample in the isothermal crystallization from the melt. The IR spectral change tells us about the formation of hydrogen bonds among the amide groups. The N–H stretching band at 3450 cm^−1^ originates from the amide groups without any hydrogen bonds, the intensity of which decreased after the temperature jump (time region A). At the same time, the hydrogen-bonded NH band at 3320 cm^−1^ increased in intensity. Therefore, it may be said that, even in the molten state, a number of hydrogen bonds were formed in addition to the free amide groups. This observation suggests the existence of the domains composed of the nylon chain segments connected by the weak hydrogen bonds, though the methylene parts were totally disordered. Correspondingly, the SAXS data shows the central scattering from the early stage of the temperature jump, which was interpreted using the Debye–Buche equation (Equation (4)) under the assumption of the existence of domains in the time region A, as indicated by the IR spectral data. In the time region B, the correlation length *ξ* among the domains was found to decrease gradually. In the time region C, the SAXS invariant *Q* started to increase and the WAXD crystalline peaks were also detected, indicating the formation of crystalline lamellae. The long period was about 200 Å at the initial stage of the region C, which was close to the correlation length *ξ* in the last stage of the region B.

The IR data tell us about the conformational regularization of the methylene segmental parts. When the time was 10~40 s after the jump (region B), the hydrogen bonds became stronger (as seen from the lower frequency shift of hydrogen-bonded NH stretching band) and the population of hydrogen bonds increased further (as known from the intensity increment). In the time region C, the slight increase in the hydrogen bonds was detected and, at this moment, the vibrational bands intrinsic to the zigzag methylene segments of CO side, –CO(CD_2_)_8_CO–, started to appear. However, the regular methylene bands of the NH side, –NH(CH_2_)_10_NH–, were not detected. This indicates that the nylon chains started to extend and form the parallel array in the crystal lattice, where only the CO-side methylene segments were regularized, but the NH-side methylene parts were still in the disordered state.

**Figure 14:**
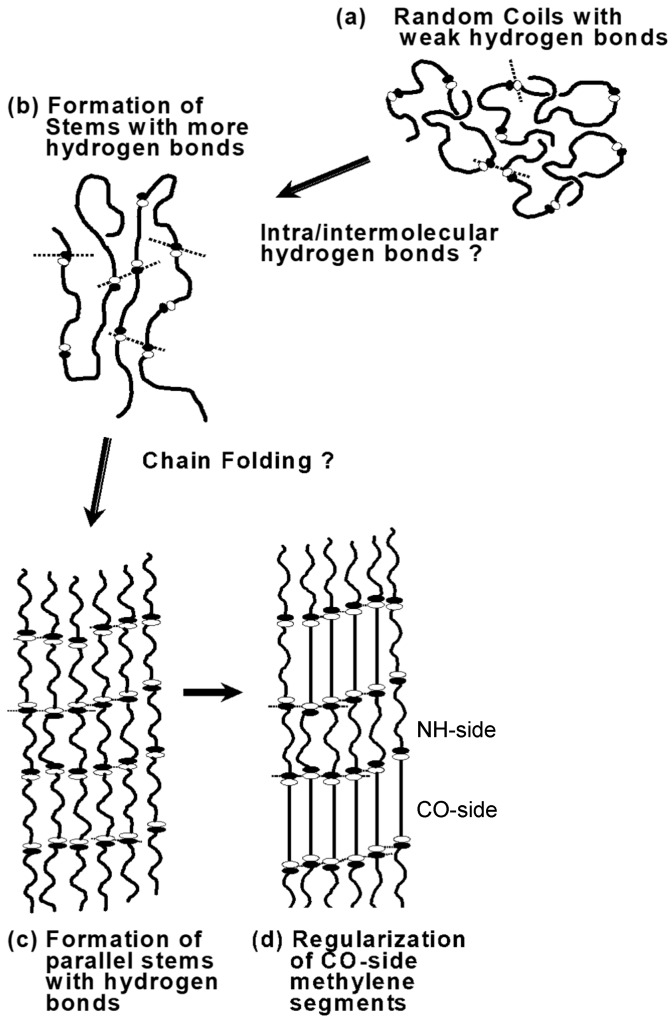
Schematic illustration of the structural evolution of nylon 1010 in the isothermal crystallization process from the melt.

As illustrated in Figure 14, nylon 1010 shows the unique structural evolution in such a way that the hydrogen bonds were already formed in the molten state, and the correlation among the domains constructed by the hydrogen-bonded chain segments became stronger with the increasing number of hydrogen-bonded amide groups (time regions A and B). Following this, the regularization of the methylene segmental parts started to occur in parallel to the formation of the crystal lattice as well as the creation of the stacked lamellar structure (time region C). It should be noted that the methylene segments of CO-side and NH-side behave in a different way in the regularization process.

### 3.6. VDF–TrFE Copolymers

**Figure 15:**
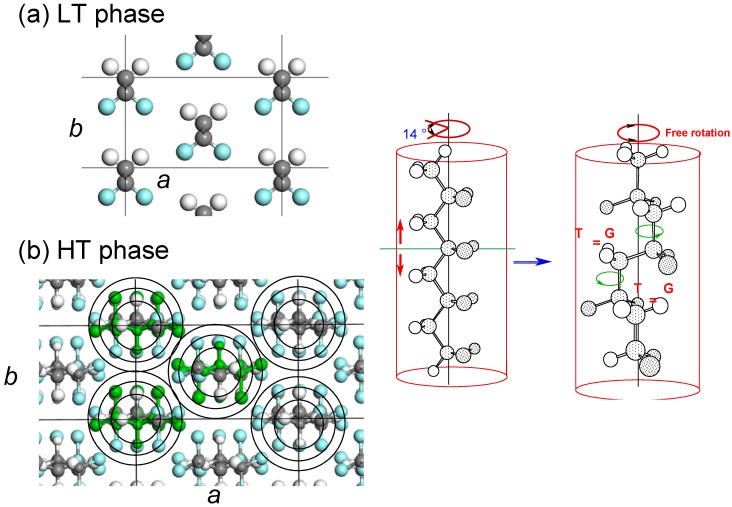
Crystal structure and chain conformation of the VDF–TrFE copolymer: (**a**) low-temperature phase and (**b**) high-temperature phase. The unit cell parameters were *a* = 9.11 Å, *b* = 5.25 Å, *c* = 2.55 Å for the LT phase and *a* = 9.75 Å, *b* = 5.63 Å, and *c* = 9.20 Å for the HT phase (VDF 55%) [43].

**Figure 16:**
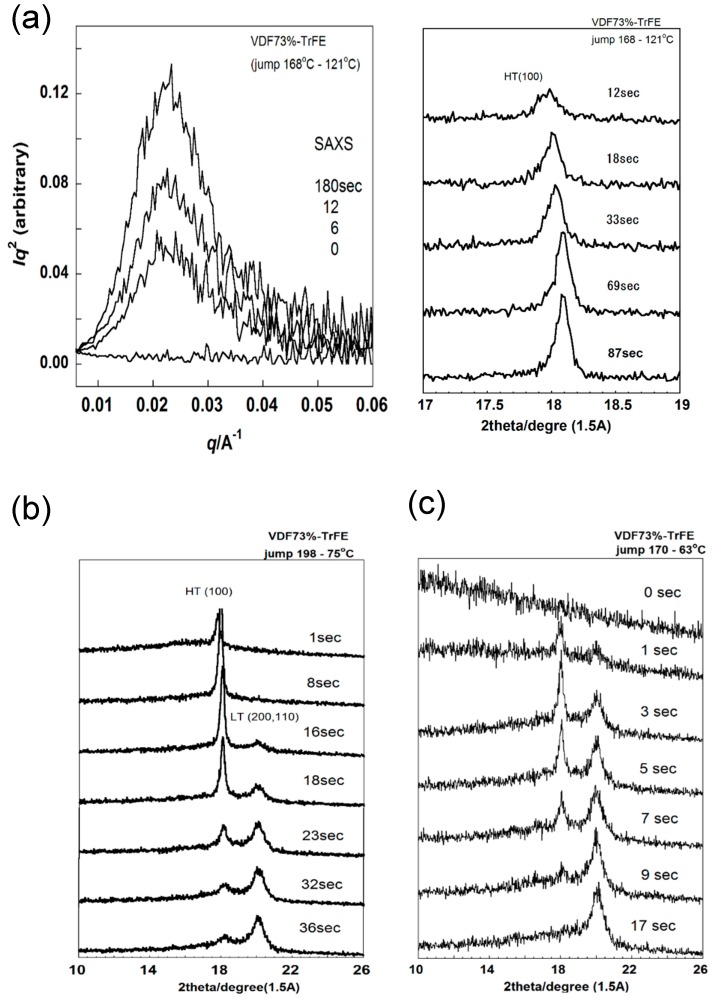
Isothermal melt-crystallization of VDF73%–TrFE copolymer: the temperature jump (**a**) to the HT phase region, (**b**) to the coexistent region of the HT and LT phases, and (**c**) to the LT phase region.

This copolymer shows the solid-state phase transition between the ferroelectric phase (the low-temperature phase, LT) and the paraelectric phase (the high-temperature phase, HT) at a Curie transition temperature (see Figure 15) [43]. In the cooling process from the melt, the HT phase crystallizes first and then transforms to the LT phase. If the sample is cooled from the molten state to the temperature region of the LT phase, the LT phase should appear directly from the melt. But we did not know whether this prediction was correct or not, and then the temperature jump experiment was performed [44]. The sample used was a copolymer of vinylidene fluoride (VDF) 73 mol % with trifluoroethylene (TrFE) 27 mol % (abbreviated as VDF73 copolymer). For example, Figure 16a shows the WAXD and SAXS pattern changes in the temperature jump from the melt (about 160 °C) to 121 °C or into the temperature region of the HT phase. In the early stage of the temperature jump, because of too strong parasitic scattering, the SAXS intensity change in the low *q* range could not be traced, but the long period peak of about 290 Å was detected. Correspondingly, the WAXD (100) reflection peak characteristic of the HT phase was detected in the same time region. The peak position of the (100) reflection was found to shift toward the higher 2θ side, indicating an occurrence of more compact packing of the chains, though the chain conformation was more or less disordered in the HT phase. When the isothermal melt-crystallization was performed at a lower temperature (75 °C), or into the transition region from HT to LT phase, the WAXD peak of the HT phase appeared at first, as shown in Figure 16b. After that, the LT phase peak started to increase the intensity instead of the HT phase peak. When the temperature jump was performed into a lower temperature region of the LT phase (63 °C), both of the peaks intrinsic to the HT and LT phases started to appear and coexisted for a while, and then the HT phase peak finally disappeared. In this way, the HT phase always appeared at first even when the isothermal crystallization was performed in the LT phase region. The coexistence of the two phases in Figure 16c looks curious at first glance, since the thermodynamically preferable phase at 63 °C should be the LT phase. The appearance of the thermodynamically unstable HT phase was due to the kinetic factor; the HT phase is kinetically more preferable than the LT phase. The HT phase takes the loose packing structure of the conformationally disordered chains and so this phase appears more easily than the LT phase of the regular zigzag conformation. This situation is similar to the case of the crystallization of the orthorhombic PE, where the hexagonal phase, corresponding to the HT phase, appears at first and transforms to the orthorhombic phase gradually since the latter is always thermodynamically more stable than the former as long as the crystallization is performed under atmospheric pressure. At high pressure, the hexagonal phase is existent as a stable state. In this way, the crystalline phase existent in the lamellae is controlled by a combination of kinetic and thermodynamic factors. It is dangerous to simply expect that only the thermodynamically stable phase (LT phase) appears when the crystallization experiment is performed in the low temperature region.

### 3.7. Poly(Alkylene Terephthalate)

**Figure 17:**
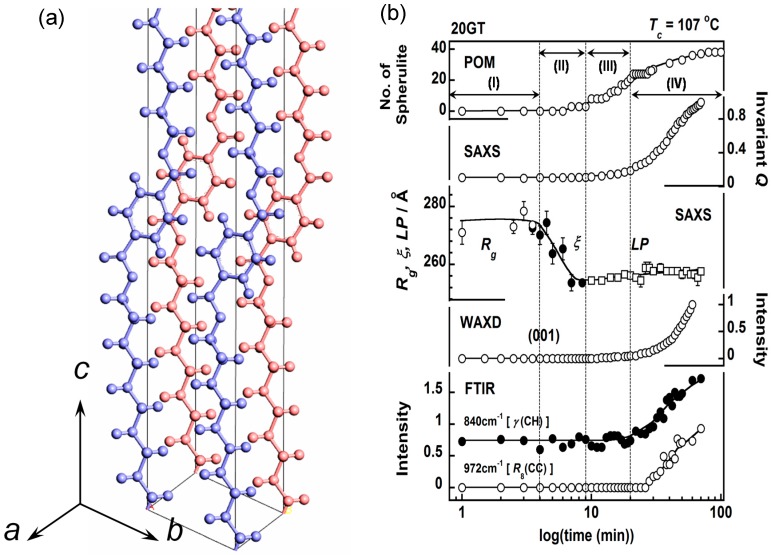
(**a**) Crystal structure of 20GT at room temperature and (**b**) the time dependence of the various data measured for 20GT in the isothermal crystallization from the melt [47].

Arylate polyester –(–O(CH_2_)*_m_*OCOФCO–)*_n_*– (PAmT or mGT) has a long methylene sequence sandwiched between the terephthalate units (OCOФCOO). Depending on the number *m* of the methylene units, the chain conformation in the crystal lattice changes systematically. In the cases of short *m* value, the chain conformation is remarkably different between the members of odd and even *m* values. PET (2GT) takes a trans-zigzag conformation [45], while 3GT takes a helical conformation contracted from the extended form by the introduction of gauche CC bonds [46]. As the number *m* is longer, the methylene segmental part tends to take a fully extended all-trans conformation irrespective of the *m* value [47,48]. In the present study, 20GT, –(–OCO–C_6_H_4_–COO–(CH_2_)_20_–)–, was targeted to study the structural regularization process in the isothermal crystallization from the melt. The crystal structure of 20GT was determined by the WAXD data analysis, as shown in Figure 17a [47]. The regularization of the long methylene units can be traced clearly by measuring the time dependent IR spectral data, since the assignment of a series of methylene progression bands was made already. The crystallization experiment was performed using a synchrotron X-ray beam in the beamline SAXS-4C of the Pohang Accelerator Laboratory (PAL), Korea. The experiments were performed with the two different setups: (i) the simultaneous FTIR/SAXS measurement and (ii) the simultaneous FTIR/WAXD measurement. The wavelength of an incident X-ray beam was 0.675 Å. The thus collected WAXD, SAXS, and FTIR spectral data were quantitatively analyzed and combined together as shown in Figure 17b. This combination can be made reasonably because the temperature jump process was essentially the same between these two independent experiments of SAXS/FTIR and WAXD/FTIR within the experimental error. In this figure, the total number of spherulites generated in the isothermal crystallization process was also plotted, which was estimated by the polarized optical microscopic observation under the same temperature jump condition. In the time region (I), after the sample temperature reached *T*_c_ at 0 min, the domains with *R_g_* of circa 275 Å, as estimated by SAXS analysis, were detected. In the time region (II), the correlation length *ξ* was almost the same as *R_g_*, and it decreased almost linearly to approach the long period of the stacked lamellae. 

In the polarized optical microscopic observation, a few numbers of tiny spherulites started to appear. The crystallization-sensitive infrared band of the CH out-of-plane deformation mode of benzene ring (*γ*(CH)) was not yet detected there. In the time region (III), the number of spherulites increased further. The X-ray 001 peak, intrinsic to the chain conformation, started to increase the intensity in this region, although not very strongly. The SAXS invariant *Q* and long-period *LP* started to be detected in parallel. The *hk*0 reflections were not detected. The methylene progression IR band (972 cm^−1^, CC stretching mode R_s_(CC)) was not detected also during this timing. In this way, in the time region (III), the stacked lamellae were formed, but the molecular chains were still in the conformationally disordered state. It is noted that the spherulites were already produced even in such a structurally disordered inner state. In other words, the spherulite was in a liquid–crystalline state. In the time region (IV), the SAXS invariant *Q*, the WAXD intensity *I*_001_, IR band intensity (*γ*(CH) and R_s_(CC)) remarkably increased in parallel, indicating the creation of a crystal structure of regularly packed chains inside the spherulites. It is important to note again that the spherulites were formed in the relatively early stage of crystallization, but the inner structure was in the disordered state, and, then, the inner structure was regularized gradually without a drastic increase in the spherulites themselves [49].

## 4. Interpretation

In this way, we observed the structural evolution in the isothermal crystallization process from the melt for the various kinds of crystalline polymers on the basis of the time-resolved measurements of WAXD, SAXS, and FTIR data. The combination of these data has allowed us to deduce the hierarchical structural changes occurring in the crystallization process in detail.

As shown in Figure 5, Figure 6, and Figure 14, the structural evolution occurred in the following way:

(i) The thermally-induced fluctuation in the melt [50] caused the heterogeneous distribution of the density and the domains were formed, which were the aggregation of the more or less conformationally disordered chain segments.
(ii) The domains became larger and increased in number. As a result, the correlation became stronger among the neighboring domains.
(iii) The domains fused into large lamellae. In these lamellae, the chain segments were arrayed in parallel, but they were not necessarily totally ordered. For the polymer with long methylene segments, the methylene conformation was still disordered more or less. In the case of nylon, the molecular chain stems in the early stage of lamellar formation possessed the regular methylene segments, but this was only in the CO-side, and the methylene segments in the NH side were still in the disordered state, although the intermolecular hydrogen bonds were already created.
(iv) The size of thus created lamellae increased with time. The random coils in the melt approached the front surface of the growing lamella and they were adsorbed into the lamellar inside to become the stems with the folded parts.
(v) The thus adsorbed and regularized stems were existent in the various states. For example, as seen in the case of VDF–TrFE copolymers, the newly attached stems were in the HT phase and coexisted with the LT phase region, or they transform totally to the more regular structure of the LT phase. It depends on the crystallization temperature. In the case of PE, the attached segments may be in the conformationally disordered trans form and exist in the hexagonal lattice for a while. This hexagonal phase is unstable and transforms to the orthorhombic phase, since only the latter is thermodynamically possible at atmospheric pressure, different from the case of VDF–TrFE copolymer. In the case of *it*-PP and POM, the random coils adsorbed on the lamellar surface changed immediately to the regular helices because of the relatively rigid helices.

As mentioned in the data analysis procedure, the SAXS data analysis was performed assuming the formation of domains, the correlation of domains, and the existence of lamellae. In such a sense, the derived structural image might be only artificial: the structural images might have been set before the SAXS data analysis. However, this is not true. We need to emphasize strongly that (a) the good fittings of the SAXS data were obtained reasonably and rationally using the theories developed for these three individual structural models; (b) at the starting point we did not know the systematic relation among these three parameters, but they showed the surprisingly good correlation after the independent data treatments; and (c) these structural concepts were supported reasonably and consistently by the quantitative analyses of IR spectroscopic data as well as the WAXD data.

The image of the lamellar growing process proposed by Strobl et al. [20,21] corresponds to the case of PE [20,21]. It is not necessarily correct to apply their model easily to the other types of polymers, since the internal state of the growing lamellae and their existing mode are different depending on the type of polymer in question. Sometimes the lamella consists of the coexistence of the two phases (as seen for VDF–TrFE copolymer), and sometimes they are still in the thermodynamically unstable HT phase as a whole, and sometimes they might be coexistent in the equilibrium state among the two crystalline regions. Figure 11 illustrates these situations schematically.

## 5. Conclusions

In the present paper, we summarized the isothermal melt-crystallization behavior observed for various types of crystalline polymers, from which the universality and individuality were extracted regarding the hierarchical structural evolution process in the isothermal crystallization phenomenon. The intermediate phase is an ambiguous concept to describe the transient state before the appearance of the regular crystalline phase. In some cases, the aggregation of the domains consisting of the short helical chain stems corresponded to the intermediate phase (*it*-PP, POM, etc.). In other cases, the whole crystalline phase was governed by the parallel array of the conformationally disordered chain stems (VDF copolymer). We must draw the image of the intermediate phase with care. Even for the same polymer species, the structural evolution behavior might be sensitively affected by a slight change in such an external crystallization condition as temperature, pressure, temperature jumping rate, etc. In order to understand the crystallization behavior more concretely, we need to know the detailed thermodynamic phase diagram and the kinetic character for the individual polymer species in addition to the structural evolution images described here.
